# Supplementary material for: Risk of Acute Anterior Uveitis in Ankylosing Spondylitis According to the Type of Tumor Necrosis Factor-Alpha Inhibitor and History of Uveitis: A Nationwide Population-Based Study
Source: J Clin Med. 2022 Jan 26;11(3):631. doi: 10.3390/jcm11030631 (PMC8836742; doi:10.3390/jcm11030631)
Supplement: Supplementary file 1 [file jcm-11-00631-s001.zip › jcm-1568127-supplementary.pdf]

Table S1. Operational definitions of inclusion/exclusion criteria, outcome, and comorbidities.

Category	Disease	ICD-10 codes	Number of diagnosis/additional definition
Inclusion	Ankylosing spondylitis	M45	V140 (rare incurable disease registration code)
Exclusion	Systemic lupus erythematosus	M32	
	Rheumatoid arthritis	M05, M06	V223 (rare incurable disease registration code)
	Bechet's disease	M352	
	Psoriatic arthritis	M07	
	Inflammatory bowel disease	K51, K50	
Outcome	Acute anterior uveitis	H200, H201, H208, H209, H221	with prescription of cyclosporin or steroids (oral/injection/topical)
Comorbidities	Charlson comorbidity index		ref. Quan 2005
	Dyslipidemia	E78	Admission or outpatient clinic ≥ 2 within a year
	Hypertension	I10-I13, I15	Admission or outpatient clinic ≥ 2 within a year
	Diabetes Mellitus	E10-E14	Admission or outpatient clinic ≥ 2 within a year
	Chronic obstructive pulmonary disease	J45-46	Admission or outpatient clinic ≥ 2 within a year
	Ischemic heart disease	I20-25	Admission or outpatient clinic ≥ 2 within a year
	Psoriasis	L40	Admission or outpatient clinic ≥ 2 within a year
	Stroke	I63, I64, I693, I694, G459	Admission or outpatient clinic ≥ 2 within a year
	Renal failure	N18, N19	Admission or outpatient clinic ≥ 2 within a year
	Asthma	J44	Admission or outpatient clinic ≥ 2 within a year

ICD-10: Tenth revision of International Statistical Classification of Diseases and Related Health Problems

Table S2. Standardized codes of Korean National Health Insurance claims database for medical treatments used in this study.

Category	Therapeutic class (Prescription duration)	General name	HIRA formulary code (4 digits)	Additional definition
Exposure	TNFis (Continuous use ≥730 days)	Adalimumab	4884	Anti-TNF-α monoclonal antibodies
		Etanercept	4558	TNF receptor
		Infliximab	3835	Anti-TNF-α monoclonal antibodies
		Golimumab	6212	Anti-TNF-α monoclonal antibodies
Co-medications	Immune modulating agents (Ever use after TNFis initiation)	Sulfasalazine	2328	
		Methotrexate	1921	
		Cyclosporine	1392, 1947, 4751	
		Azathioprine	1124	
		Mycophenolate mofetil	1978	
		Cyclophosphamide	1390	
	Use of steroid (for oral medications, ≥90 days after TNFis initiation)	Betamethasone	1164, 1165, 2969, 3161	oral/injection
		Deflazacort	1408	oral
		Dexamethasone	1419, 1420, 1422	oral/injection
		Fludrocortisone	1602	oral
		Fluorometholone	1612, 1613, 3322, 3323, 5254, 5387, 5388, 5389, 6405, 6422, 6431, 6440, 6472, 6474, 6475	topical
		Hydrocortisone	1709, 1712	oral/injection
		Methylprednisolone	1933, 1935, 1936	oral/injection
		Prednisolone	2170, 2171, 2172, 2173, 2174, 2175, 3617	oral/injection/topical
		Rimexolone	2241	topical
		Triamcinolone	2432, 2433	oral/injection
	Use of NSAIDs (for oral medications ≥0.7 PDC during TNFis treatment)	Aceclofenac	1009	
		Acemetacin	1010	
		Celecoxib	3477	
		Cinnoxicam	1338	

Clonixin	1366
Dexibuprofen	1423
Dexketoprofen	4543, 6710
Diclofenac	1432, 1433, 1434, 1435, 1436, 2519, 4828
Etodolac	1566
Etoricoxib	6369
Fenoprofen	1578
Flufenamic acid	1603, 3407
Flurbiprofen	1619, 1620
Ibuprofen	1728, 1729, 3133, 3134, 3636, 3782, 3842, 4409, 4415, 4479, 4510, 4566, 6014, 6699, 6852
Ibuproxam	1729
Ketoprofen	1797, 1798, 1799, 4265, 4543
Ketorolac	1800
Lornoxicam	3555
Loxoprofen	1861
Mefenamic acid	1890
Meloxicam	1897, 4271
Morniflumate	4584
Nabumetone	1981
Naproxen	1994, 1995, 4503
Nimesulide	2018
Pelubiprofen	4934
Piroxicam	2140, 2141, 2142
Polmacoxib	6364
Pranoprofen	2165
Proglumetacin	2186
Sulindac	2332
Talniflumate	2344
Tenoxicam	2353
Tiaprofenic acid	2389
Tolfenamic acid	2415
Zaltoprofen	2499

HIRA: Health Insurance Review and Assessment; TNFis: tumor necrosis factor (TNF)-alpha inhibitors; NSAIDs: non-steroidal anti-inflammatory drugs; PDC: proportion of days covered.
